# Ability to perform activities of daily living is the main factor affecting quality of life in patients with dementia

**DOI:** 10.1186/1477-7525-2-52

**Published:** 2004-09-21

**Authors:** Christian K Andersen, Kim U Wittrup-Jensen, Anette Lolk, Kjeld Andersen, Per Kragh-Sørensen

**Affiliations:** 1 MUUSMANN Research & Consulting, Haderslevvej 36, 6000 Kolding, Denmark; 2 Global Health Economics & Outcomes Research, Novo Nordisk A/S 2880 Bagsværd, Denmark; 3 Center for Dementia Research, Odense University Hospital, Sdr. Boulevard 29 5000 Odense, Denmark

## Abstract

**Background:**

Dementia is a chronic illness associated with a progressive loss of cognitive and intellectual abilities, such as memory, judgment and abstract thinking.

The objective of this study was to assess the health utilities of patients with dementia in Europe and identify the key factors influencing their Health-Related Quality of Life (HRQol).

**Methods:**

This study used cross-sectional data from the Odense study; a Danish cohort of patients aged 65–84 living in Odense, Denmark. A total of 244 patients with mild to severe dementia were interviewed together with a caregiver about their health status and activities of daily living (ADL). Alzheimer's disease was diagnosed according to the NINCDS-ADRDA criteria for probable dementia. Vascular dementia and other types of dementia were diagnosed according to the DSM-IIIR criteria. Severity of dementia was defined by score intervals on the Mini Mental State Examination score: mild (MMSE 20–30), moderate (MMSE 10–19), and severe (MMSE 0–9). Based on the ADL information, the patients' dependency level was defined as either dependent or independent. Questions from the Odense Study were mapped into each of the five dimensions of the EQ-5D in order to assess patients' HRQol. Danish EQ-5D social tariffs were used to value patients' HRQol.

A regression analysis of EQ-5D values was conducted with backward selection on gender, age, severity, ADL level and setting in order to determine the main factor influencing HRQoL.

**Results:**

The EQ-5D weight in patients independent upon others in ADL was 0.641 (95% CI: [0.612–0.669]), and in those dependent upon others was 0.343 (95% CI: [0.251–0.436]).

**Conclusion:**

Dependency upon others to perform ADL was the main factor affecting HRQoL.

## Background

Dementia is a chronic illness associated with a progressive loss of cognitive and intellectual abilities, such as memory, judgment and abstract thinking. Cognitive disabilities are those that impact an individual's ability to access, process, or remember information. People with profound cognitive disability will need assistance with nearly every aspect of daily living. The most visible manifestation of dementia is the progressive inability – proportional to the severity of the disease – to perform activities of daily living (ADL) and the subsequent loss of independence [1]. Progressive deterioration in the cognitive, functional and behavioural domains eventually brings patients to the later stages of dependency and, in most cases, to institutionalisation, which is linked to an increased need in caregiver assistance [2]. A patient's level of dependency is a global measurement reflecting a certain level of severity, resource consumption and Quality-of-Life (QoL) [3].

Measuring the QoL of patients suffering from dementia can take several forms. Firstly, QoL can be measured using generic health indices like the other disease specific measures. Recently several scales have been developed and validated specifically for dementia patients such as the Quality of Life-Alzheimer's Disease (QOL-AD). Another alternative to assess QoL is to use utility measurements, which are preference-based [4]. Preference-based measures evaluate the patient's preference for a health state instead of measuring the frequency and the severity of symptoms or disabilities. In order to use quality-adjusted life years (QALYs) as outcome measures for cost-effectiveness analyses, utility-weighted measures of Health-Related Quality-of-Life (HRQoL) are required. These attribute a single number to a health state using a common unit of measure allowing comparison between different strategies [5]. In general, however, HRQoL is not as broad a concept as QoL. One of the more reliable and newer tools used to measure HRQoL in a wide range of health conditions and treatments including dementia is the EQ-5D [6]. It is a generic measure designed to complement disease specific outcome measures and health characterises on five dimensions: mobility, self-care, ability to perform usual activities, pain, and anxiety/depression. It provides a descriptive health profile and a single index value for health status and, as such, it can be used to estimate utility in pharmacoeconomic evaluations of new pharmacological treatments.

Health utilities have already been measured in AD in the US and Canada using the Health Utility Index (HUI) [7]. In the UK, the EuroQol instrument has been used to investigate whether HRQol data could be obtained from proxies, such as family caregivers [8]. In France, the EuroQol instrument has been administered to patients with dementia in order to determine the feasibility, reliability, and validity of the French version of the EuroQol instrument [9]. However, health utilities are not reported from the two latter studies. Furthermore, HRQol data are needed in order to carry out cost-effectiveness analysis for a Danish setting.

Based on data collected alongside an epidemiological study conducted in Odense Denmark, we attempted to assess the health utilities of patients with dementia and identify the key factors influencing their HRQoL.

## Methods

### Population

Data were derived from the Odense study, an epidemiological survey in which the objective was to estimate the prevalence and incidence of dementia in Denmark [10,11] In this study, a total of 244 patients with dementia agreed to participate in an interview accompanied by a relative or caregiver. The study was approved by the Scientific-Ethical Committee of the Counties of Funen and Vejle, Denmark, and by the Danish Data Protection Agency.

Demented patients were classified by type of dementia and by severity of dementia. Alzheimer's disease (AD) was diagnosed according to the NINCDS-ADRDA criteria for probable dementia [12]. Vascular dementia and other types of dementia were diagnosed according to the DSM-IIIR criteria [13]. Severity of dementia was diagnosed according to the Clinical Dementia Rating (CDR) scale [14] and the Mini Mental State Examination (MMSE) [15]. The complete examination programme is described in Andersen, Lolk et al, 1997 [10].

### Assessments

All interviews were conducted by a certified nurse in the patient's home., Patient's and caregiver's socio-economic and socio-demographic status as well patients' health status and ADL were recorded. In the event that a relative was not present during the interview, a professional caregiver verified information provided by the patient.

Each interview included the following information:

- sociodemographic questions (age, gender, setting).

- activity of daily living (ADL) questionnaire using 7 items describing patients' ability to perform physical activities (personal care, dressing, mobility and personal toiletry) and psychosocial activities (activities in the home and hobbies inside and outside of the home). Each activity was scored using a four-point Likert scale anchored at the ends with 1 = "Unable to perform the activity" and 4 = "Perform the activity without help from others". The physical ADL ranged between 4 (worst state) and 16 (best state), while the psychosocial ADL scored between 3 (worst state) and 12 (best state) [16].

- Questions mapped into each of the five dimensions of the EQ-5D: mobility, self-care, usual activities, pain/discomfort, and anxiety/depression as presented in the Table 1[17].

**Table 1:** Mapping questions from the Odense Study into the EQ-5D

**EQ-5D**	**The Odense Study**
**Mobility**	**Mobility**
1. I have no problems in walking around.	• Without help from others.
2. I have some problems in walking around.	• Needs some help from others.
	• Needs help from others.
3. I am confined to bed.	• Unable to walk around without help.
**Personal care**	**Personal care/dressing**
1. I have no problems with self-care.	• Without help from others.
2. I have some problems with self-care.	• Needs some help from others.
	• Needs help from others.
3. I am unable to wash or dress myself.	• Unable to wash or dress without help.
**Usual activities**	**Hobbies in the home**
1. I have no problems with performing my usual activities.	• Without help from others.
2. I have some problems with performing my usual activities.	• Needs some help from others.
	• Needs help from others.
3. I an unable to perform my usual activities.	• Unable to perform hobbies in the home.
**Pain/discomfort**	**Patient's assessment of own health status**
1. I have no pain or discomfort.	• Very good.
2. I have moderate pain or discomfort.	• Good/fair.
3. I have extreme pain or discomfort.	• Poor.
**Anxiety/depression**	**Patient's experience of emotional problems**
1. I am not anxious or depressed.	• Never.
2. I am moderately anxious or depressed.	• Sometimes.
3. I am extremely anxious or depressed.	• Often.

For the mobility dimension of the EuroQol instrument, we assumed that patients that were able to walk without assistance from others had no problems in performing this activity, whereas patients who were unable to walk unassisted were classified as confined to bed. Patients that needed help from others were classified as having some mobility problems.

Two questions from the ADL instrument in the Odense Study were used to classify patients on the EuroQol personal care dimension. Patients that performed both activities without help from others were classified as having no problems on this dimension. Patients in need of help with either personal care or dressing or both were classified as having some problems. Only patients unable to wash and dress without help from others were classified as such on the EuroQol instrument.

The patient's ability to carry out hobbies in the home was used as a proxy for their ability to perform usual activities.

For the pain/discomfort dimension of the EuroQol instrument, it was assumed that patient assessment of their own health status covered this dimension. Therefore, if they found their health status to be very good they were classified as having no pain or discomfort. A good or fair assessment was categorised as having moderate pain or discomfort, whereas a poor assessment was assumed to correspond to extreme pain or discomfort.

In the Odense Study, patients stated how often they experienced emotional problems, whereas the health state being described by the EuroQol instrument refers to the patient's health at the time of filling in the instrument. Thus, the questions in the Odense Study included an aspect of time that the EuroQol does not cover. To overcome this, it was assumed that the occurrence of emotional problems converts to the degree of anxiety or depressions. That is, patients that never experience emotional problems were assumed not to be anxious or depressed, whereas patients that sometimes or often experience problems converted to moderate or extreme anxiety or depression, respectively.

The procedure of mapping returned a five-digit code, where the first digit referred to the patient's mobility level; the second to the patient's level on personal care; and so forth. This five-digit code described a health state for which we looked up the HRQoL utility weight in a table of EQ-5D tariffs. The EQ-5D tariffs take values between zero and one, where zero is the worst imaginable health status, and one is the best imaginable health status. We used Danish EQ-5D tariffs from a survey based on the time trade-off technique [18].

### Patients classification

Based on their cognitive and functional scores patients were classified by severity and dependency level.

### Severity Status

The severity of a patient's dementia was defined by score intervals on the MMSE [15]. Those scoring ≥20 were considered as having mild dementia, while patients scoring between 10 and 19 were classified as having moderate dementia. Patients scoring ≤9 were classified as suffering from severe dementia. MMSE scores were not available for 30 patients (21 AD patients and 9 patients suffering from vascular dementia). In order to determine these patients degree of dementia we used the CDR score to classify them into the above three severity groups. Patients with a CDR score of 0.5 were classified as mild, patients with a CDR of 1 were classified as moderate and patients with a CDR of 2 to 3 were classified as severe [19].

### Dependency Status

Patients were classified by their ability to perform physical and psychosocial activities of daily living (ADL) This resulted in a classification of either dependent or independent [3]. A binary variable was based on a non-hierarchical cluster analysis [20]. Firstly, we identified possible initial seeds for the analysis. The seeds were identified from a cross table of the physical ADL score and the psychosocial ADL score. Combinations of the physical and psychosocial ADL scores with five or more observations were included in the cluster analysis as possible seeds. Secondly, we carried out the cluster analysis using the PROC FASTCLUS procedure in SAS 8.2 (SAS Institute Inc., Cary, NC, USA) in order to identify two clusters.

One cluster included patients with low scores on both the physical and psychosocial ADL scales. As low scores on both instruments meant that patients required help from others in performing the activities in question, they were classified as "Dependent". The other cluster included patients with high scores on both ADL scales and they were classified as "Independent". As these were composite criteria, the characteristics of the two groups of dependency were analysed.

### Statistical Analysis

After analysing the descriptive results of the EQ-5D scores, we performed a regression analysis of EQ-5D scores on sociodemographic and clinical characteristics. Sociodemographic characteristics included gender, age and setting (living in the community or institutionalised) and clinical characteristics took into account the level of severity (Mild, Moderate, and Severe), the type of dementia (AD, vascular or other) and ADL status (independent or dependent).

The regression analysis was performed with backward selection (level 5%) in order to determine the main factors influencing QoL.

Because of heteroscedasticity, we estimated the heteroscedasticity consistent covariance matrix, which was used to calculate test statistics for the coefficients.

Observations with missing data were automatically excluded from the analyses. That is, observations with insufficient information to establish a EQ-5D weight, e.g. that information was lacking to determine a patient's mobility level on the EQ-5D instrument.

## Results

Table 2 illustrates the characteristics of patients included in the Odense Study by type of dementia. Of the 244 patients, 164 (67%) suffered from Alzheimer's disease (AD) and 80 suffered from vascular or other types of dementia. On average, AD patients were 3.9 years younger (95% confidence interval: [2.5 – 5.3]) than patients suffering from vascular or other types of dementia and more AD patients lived in a nursing home (p = 0.03). The remainder of patient characteristics in Table 2 did not significantly differ between the two groups.

**Table 2:** Background Characteristics of Patients in the Odense Study

	Patients suffering from Alzheimer's disease	Patients suffering from vascular or other types of dementia	All patients suffering from dementia
Number of patients	164 (67.2%)	80 (32.8%)	244
Number of females	89 (54.3%)	33 (41.3%)	122 (50.0%)
Mean (SD) age in years	79.4 (5.14)	75.5 (5.21)	78.1 (5.47)
Mean (SD) MMSE score	20.6 (4.62)	21.6 (4.96)	21.0 (4.75)
Severity of dementia			
Mild (MMSE 20–30)	91 (55.5%)	49 (61.3%)	140 (57.4%)
Moderate (MMSE 10–19)	51 (31.1%)	23 (28.8%)	74 (30.4%)
Severe (MMSE 0–9)	22 (13.4%)	8 (10.0%)	30 (12.3%)
Mean (SD) physical ADL score	14.1 (3.08)	13.7 (2.96)	14.0 (3.04)
Mean (SD) psychosocial ADL score	9.3 (2.81)	9.0 (2.39)	9.2 (2.68)
Living in the community	132 (80.5%)	73 (91.3%)	205 (84.0%)

Tables 3 and 4 show the results from the cluster analysis, which was used to classify patients, according to their ADL status, as either independent or dependent in performing activities of daily living. Table 3 presents cluster characteristics. Of the 244 patients with dementia, 38 (16%) were classified as dependent, and 206 (84%) were classified as independent in the performance of ADL. Dependent patients were, on average, more severely stricken and institutionalised than independent patients.

**Table 3:** Cluster Description

	**Dependent**	**Independent**	**p-value**
Number of patients	38 (16%)	206 (85%)	
Number of females	17 (45%)	105 (51%)	0.4801
Mean (SD) age in years	79.3 (5)	77.9 (5)	0.1493
Number of AD patients	24 (63%)	140 (68%)	0.5622
Mean (SD) MMSE score	16.8 (6)	21.4 (4)	<0.0001
Mean (SD) physical ADL score	7.9 (2)	15.1 (1)	<0.0001
Mean (SD) psychosocial ADL score	4.7 (2)	10.1 (2)	<0.0001
Number of patients living in the community	16 (42%)	189 (92%)	<0.0001

**Table 4:** Number of Patients by Dependency Status and Type of Dementia

**ADL status**	**Patients suffering from Alzheimer's disease**		**Patients suffering from vascular or other types of dementia**		**All**	
Dependent	24	(15%)	14	(18%)	38	(16%)
Independent	140	(85%)	66	(83%)	206	(84%)
All	164	(100%)	80	(100%)	244	(100%)

Table 4 shows the number of patients by dependency status and type of dementia. Due to of missing data, EQ-5D values were estimated for only 211 patients upon 244 demented patients. Results of comparison of EQ-5D value in different subgroups of patients were shown in Table 5. Patient QoL seems to decrease when severity and dependency increase as well as when patients are institutionalised.

**Table 5:** Mean EQ-5D weights by patient subgroups

	**N**	**Mean**	**SD**
**Severity Subgroups**			
Mild MMSE > 20	135	0.636	(0.2109)
Moderate 9 < MMSE < 20	64	0.596	(0.2152)
Severe MMSE < 10	12	0.486	(0.2191)
**Dependency Subgroups**			
Independent	193	0.641	(0.1952)
Dependent	18	0.343	0.2324)
**Setting subgroups**			
Community	191	0.621	(0.2173)
Institution	20	0.564	(0.1861)

Table 6 shows the results of a regression analyses. The results from the full model that included all predictors, as well as results from the reduced model that included only significant predictors are presented.

**Table 6:** Results of the Regression Analysis of EQ-5D TTO Tariff on Severity of Dementia, ADL Status and Setting (n = 211)

	Full model			Reduced model		
Variable	Coefficient	Asymptotic standard error	p-value	Coefficient	Asymptotic standard error	p-value
Constant	0.579	0.0371	<0.0001	0.641	0.0140	<0.0001
Gender (0 = male, 1 = female)	0.045	0.0266	0.0948	-	-	-
Age	0.003	0.0029	0.3553	-	-	-
Type of dementia (0 = Other, 1 = AD)	0.006	0.0327	0.8522	-	-	-
Severity of dementia						
Moderate	0.043	0.0280	0.1258	-	-	-
Severe	-0.079	0.0534	0.1405	-	-	-
Dependency status (0 = independent, 1 = dependent)	-0.289	0.0534	<0.0001	-0.297	0.0551	<0.0001
Setting (0 = community, 1 = nursing home)	0.027	0.0368	0.4672	-	-	-
R^2^		0.1776			0.1502	

Patients dependent upon others to perform activities of daily living clearly had a lower QoL than independent patients. The QoL of independent patients suffering from dementia as assessed by their capacity to perform activities of daily living was 0.641 (95% confidence interval: [0.612 – 0.669]) whereas the QoL of dependent patients for the same assessment was 0.343 (95% CI: [0.251 – 0.436]). Severity of dementia and setting has no statistically significant impact on QoL.

## Discussion

For the first time, this study provides health utilities for patients with dementia in Denmark. This study has shown that the factor that most affects the HRQoL of a patient with dementia is their dependency status as defined based by their ability to perform activities of daily living. The type of dementia doesn't seem to have a great an influence on patient's HRQoL, and severity does not appear to discriminate significantly between health utilities. However, due to missing data – particularly among patients with severe dementia – caution must be exercised when interpreting the results.

In the utility results previously measured by Neumann et al. [7] using the Health Utility Index (HUI), AD patients' utilities decreased significantly with their severity levels. However, this could be explained by the fact that the EQ-5D does not consider cognition as a separate attribute, unlike the HUI scales. Despite this difference, results obtained with the HUI and the EQ-5D instruments were within the same range.

As the EQ-5D values were estimated based on mapped questions, it raises the possibility of quotation bias and goodness of fit. Also, both patients and caregivers answered questions. Yet, with AD – and especially when patients are severely demented – it is impossible to collect non-proxy measurements in the later stages. The same methodology was performed in previous evaluations without knowing the impact of the difference between caregivers' and patients' perceptions.

A particular strength of this study was that all data have been collected in conjunction with an epidemiological study wherein patients with dementia had been examined carefully and dementia criteria were explicitly stated.

## Conclusion

Measuring HRQoL is as important as measuring disease severity, progression, symptom response, cognition and behavioural disturbance when assessing the impact of disease and determining proper intervention in the treatment and management of dementia. However, HRQoL is difficult to assess in a disease such as dementia for which patients suffer from cognitive disabilities. Based on study results and as previously shown by Kurz et al. [3], dependency level greatly influences patients' HRQoL and, when viewed as a global measure, reflects a certain level of HRQoL. Determining dependency levels could be considered as an indirect evaluation of HRQol. Other studies with disease specific questionnaires such as QoL-AD are needed to confirm these findings.

## Abbreviations

ADL Activities of Daily Living

EQ-5D EuroQol – Five Dimension Scale

EuroQol EuroQol Scale

DSM-IIIR Diagnostic and Statistical Manual of Mental Disorders 3^rd ^edition

HRQol Health-Related Quality of Life

MMSE Mini Mental State Examination

QoL Quality of Life

SAS Statistical Analytical Software

## Authors Contributions

CKA is principal author and responsible for quality control. K W-J provided mapping of the questions from the Odense Study into the five dimensions of the EQ-5D. AL and KA provided data analysis and data analyses of the Odense database. P K-S provided access to the Odense database.

## References

[B1] Potkin SG (2002). The ABC of Alzheimer's disease: ADL and improving day-to-day functioning of patients. Int Psychogeriatr.

[B2] Bullock R, Hammond G (2003). Realistic expectations: the management of severe Alzheimer disease. Alzheimer Dis Assoc Disord.

[B3] Kurz X, Scuvee-Moreau J, Rive B, Dresse A (2003). A new approach to the qualitative evaluation of functional disability in dementia. Int J Geriatr Psychiatry.

[B4] Bennett KJ, Torrance GW, Spilker B (1996). Measuring heralth state preferences and utilities: rating scale, time trade-off, and standard gamble techniques. Quality of Life and Pharmacoeconomics in Clinical Trials.

[B5] Dolan P, Drummond M, McGuire A (2001). Output measures and valuation in health. In Economic evaluation in health Care Merging theory with Practice.

[B6] Brooks R (1996). EuroQol: the current state of play. Health Policy.

[B7] Neumann PJ, Sandberg EA, Araki SS, Kuntz KM, Feeny D, Weinstein MC (2000). A comparison of HUI2 and HUI3 utility scores in Alzheimer's disease. Med Decis Making.

[B8] Coucill W, Bryan S, Bentham P, Buckley A, Laight A (2001). EQ-5D in patients with dementia: an investigation of inter-rater agreement. Med Care.

[B9] Ankri J, Beaufils B, Novella JL, Morrone I, Guillemin F, Jolly D, Ploton L, Blanchard F (2003). Use of the EQ-5D among patients suffering from dementia. J Clin Epidemiol.

[B10] Andersen K, Lolk A, Nielsen H, Andersen J, Olsen C, Kragh-Sorensen P (1997). Prevalence of very mild to severe dementia in Denmark. Acta Neurol Scand.

[B11] Andersen K, Nielsen H, Lolk A, Andersen J, Becker I, Kragh-Sørensen P (1999). Incidence of very mild to severe dementia and Alzheimer's disease in Denmark. Neurology.

[B12] McKhann G, Drachman D, Folstein M, Katzman R, Price D, Stadlan EM (1984). Clinical diagnosis of Alzheimer's disease: report of the NINCDS-ADRDA Work Group under the auspices of Department of Health and Human Services Task Force on Alzheimer's Disease. Neurology.

[B13] American Psychiatric Association (1987). Diagnostic and statistical manual of mental disorders.

[B14] Berg L (1988). Clinical Dementia Rating (CDR). Psychopharmacol Bull.

[B15] Folstein MF, Folstein SE, McHugh PR (1975). "Mini-mental state". A practical method for grading the cognitive state of patients for the clinician. J Psychiatr Res.

[B16] Salling AL (1990). Stimulation af patients aktivitie og udvikling (Stimulation of patient's activity and development. In Danish). Doctoral thesis.

[B17] Ullerup K (1999). An economic evaluation of donepezil (Aricept) for the treatment of Alzheimer dementia using a Markov cohort simulations model (In Danish).

[B18] Pedersen KM, Wittrup-Jensen KU, Brooks R, Gudex C (2003). Valuation of health. The theory of quality adjusted life years and a Danish application (In Danish).

[B19] Reisberg B, Franssen E, Souren L, Kenowski S, Auer S, Wimo A, Jönsson B, Karlsson G, Winblad B (1998). Severity scales. Health Economics of Dementia.

[B20] Sharma A (1996). Applied Multivariate Techniques.

